# Comparison of Different Techniques in Post-Extractive Socket Regeneration Using Autologous Tooth Graft: Histological and Clinical Outcomes

**DOI:** 10.1055/s-0043-1772251

**Published:** 2023-09-20

**Authors:** Elio Minetti, Andrea Palermo, Marco Berardini

**Affiliations:** 1 Department of Biomedical, Surgical, Dental Science, University of Milan, Milan, Italy; 2 College of Medicine and Dentistry, University of Birmingham, Birmingham, United Kingdom; 3 Private Practice, Pescara, Italy

**Keywords:** alveolar socket preservation, bone, bone regeneration, demineralized dentin, Tooth Transformer

## Abstract

**Objective** Post-extractive socket grafting techniques reduce alveolar ridge dimensional changes. Numerous graft materials have been suggested and a growing interest in tooth material has been observed as a valuable alternative to synthetic biomaterials or xenografts. Furthermore, different clinical procedures have been proposed for the wound closure of the post-extractive site. This study aims to compare histological and clinical outcomes of two different surgical techniques to seal the post-extractive site with the use of autologous demineralized extracted tooth as graft material.

**Materials and Methods** Sixteen post-extractive socket without buccal and/or palatal bone walls, in sixteen healthy patients, were grafted with the autologous tooth material treated by the new Tooth Transformer device (Tooth Transformer, Milan, Italy). Alveolar socket preservation procedures were performed without flap elevation. Patients were randomly subdivided into two equal groups according to the site closure technique. In group A, the pedunculate tissue was used, while in group B ice cone technique. A bone samples were collected in each site after 4 months for histological analysis.

**Results** No significant clinical differences among the different sealing techniques were observed. In both groups, the site was filled by new bone formation after 4 months of healing. The histological analysis revealed 46.1 ± 8.07% of bone volume, 9.2 ± 9.46% of residual graft, and 35.2 ± 12.36% of vital bone in group A, while group B shows 41.22 ± 5.88% of bone volume, 7.94 ± 7.54% of residual graft, and 31.7 ± 7.52% new bone. No statistical differences were detected ( *p* > 0.05).

**Conclusion** Further studies with a large number of patients, and different observation periods will be needed to confirm the results of this pilot study; however, the interesting data obtained have shown how these techniques, mixed with the autologous dentin derived graft material, seem to promote bone regeneration and reduce physiological bone resorption during alveolar socket preservation treatments.

## Introduction

Bone regeneration continues to be one of the most studied areas by researchers. Although many bone replacement materials have been suggested, autologous bone remains the gold standard in this field, 1 but the disadvantages of using autologous bone are well known (morbidity, limited amount that can be taken) and for these reasons it is necessary to use another material that may have these properties but without these limitations. 2 3 4 5 6

It is well known that, after a tooth extraction, bone remodeling phenomena lead to unpredictable resorption of hard and soft tissues 7 and the most used procedure to reduce bone loss is the alveolar socket preservation. 8

The irreversible process of three-dimensional alveolar bone resorption occurs after the tooth extraction and the average vertical reduction, after 1 year, is about1.67 mm and the horizontal volumetric reduction about 3.87 mm. 9

About the 87% of the graft materials available on the market (alloplast and xenograft) work like a scaffold for the autologous bone cells (osteoconduction) 10 but is not able to induce bone cells differentiation (osteoinduction). The tooth grafting procedure has been introduced by Yeomans and Urist more than 50 years ago, when they discovered the osteoinduction potential of demineralized dentin matrix. 11 12 More recently, Bessho et al demonstrated the presence of bone morphogenetic proteins (BMPs) in human dentin matrix. Bone formation and osteoblasts presence were observed in rat muscle after demineralized human dentin matrix graft. 13 It was also observed that the chemical composition of bone and dentin was almost the same with the presence of an inorganic portion made of hydroxyapatite and an organic one, mainly composed by collagen type 1 and other secondary proteins. 14

From this observation, in the last few years, an innovative medical device (Tooth Transformer SRL, Via Washington, 59–Milan, Italy) to obtain suitable tooth graft materials starting from the whole tooth of the patient was introduced to the market. Numerous studies have been published in the tooth graft regeneration field since this innovative device was introduced. 15 16 17

It is important to underline that the surgical approach to post-extractive socket preservation is related to the residual anatomy of the defect. For these reasons, extraction sockets have been classified, by Elian et al, into three types based on the facial/buccal bone and soft tissue position. 18 According to this simplified classification when the extraction socket presents intact soft and hard tissue is type 1, when the soft tissue is intact, but the buccal bone is partially or totally lost is type 2, while the type 3 represents the situation of total absence of both soft and hard tissues.

Chu et al, 19 in 2015, published a clinical case study presenting a new subclassification of the type 2 extraction sockets. They suggested, in fact, three new subclasses based on the precise measurement of the bone dehiscence (starting from the free gingival margin) to better describe the clinical situations in which the buccal bone is partially resorbed. For this reason, they distinguished a subclass 2A with a dehiscence defect roughly 5 to 6 mm from the free gingival margin, a subclass 2B in which the resorption involves the middle one-third of the labial plate, approximately 7 to 9 mm from the free gingival margin and a subclass 2C in which the dehiscence defect involves the apical one-third of the labial osseous plate roughly 10 mm or greater from the free gingival margin.

The aim of this clinical study is to compare clinical and histological outcomes of two different socket preservation techniques in type 2 (subclass 2B) extraction socket classification using a resorbable membrane and autologous tooth graft. These two flapless surgical approaches differ from each other in the use or not of the pedunculated tissue.

The autologous dentin graft, obtained starting from the whole extracted tooth of the patient in a few minute and chairside, may represent the present and the future “gold standard” technique to approach every situation in which a tooth extraction is needed. The main objective of fresh post-extractive socket grafting should be to obtain new bone formation and to avoid (or substantially) reduce the bone dimensional changes. It is also very important, in authors' opinion, to get new vital bone formation with the lesser possible presence of residual graft particles. For this reason, the bone regeneration should be evaluated both from a clinical and histological point of view. This study aims to evaluate the bone regeneration amount in post-extractive sockets by showing primary clinical outcomes and secondary histological outcomes. The study hypothesis is regarding the fact that if the pedunculated tissue presence could affect the bone regeneration quality or quantity in fresh post-extractive sockets, filled with autologous tooth graft material and covered by a collagen membrane.

## Materials and Methods

### Patient Selection

Subjects were recruited from healthy nonsmoker patients needing tooth extraction in the upper and lower maxillae with a class 2 defect according to Elian et al classification. All the selected dehiscence could belong to 2B subclass according to Chu et al subclassification.

For this reason, patients underwent to a preliminary cone-beam computed tomography (CBCT) scan of the area interested to measure the dehiscence of the buccal bone wall (ranging between 7 and 9 mm approximately) of the tooth needing extraction before the day of surgery. In addition, immediately after the tooth extraction the sockets bone walls were checked with a periodontal probe to confirm from a clinical point of view the vertical bone buccal bone defect between 7 and 9 mm.

All data were anonymized. This study was carried out following the principles embodied in the Helsinki Declaration, in its latter form. 20 On March 21st 2019, the University of Chieti Ethics Committee (Italy) authorized the clinical study protocol on a human model registered under the number: 638—21/3/19.

### Inclusion Criteria

- Subjects who underwent surgical intervention for tooth removal and alveolar socket preservation by using only tooth-derived bone substitute (Tooth Transformer—Tooth Transformer srl).
- Subjects who underwent implant placement in the same site of socket preservation.
- Subjects with type 2 bone walls defect (following Elian et al classification), subclass 2B (following Chu et al subclassification)
- Subjects who did not present any systemic diseases and conditions that could cause an impairment of the bone metabolism.

### Surgical Protocol

All patients received oral hygiene instructions and debridement 2 weeks before surgery. Prior to intervention, patients had to rinse with 0.2% chlorhexidine mouthwash for 1 minute (Curasept, Curaden Healthcare, Saronno, Italy). All patients received prophylactic antibiotic therapy (amoxicillin and clavulanic acid 1 g, 1 h before tooth extraction and 1 g three times/day for the next 4 days). 21 22

Treatment was performed in local anesthesia by articaine hydrochloride with epinephrine 1:100,000 (Orabloc, Pierrel, Milan, Italy).

Patient were randomly divided in two groups according to the surgical solution used to seal the post-extractive socket. Patients showing the presence of pedunculated tissue were enrolled in group A, while patients without it were enrolled in group B. The two surgical procedures proposed are as an alternative to each other depending to the presence of pediculate inflammatory tissue.

#### Group A

**Fig. 1:**
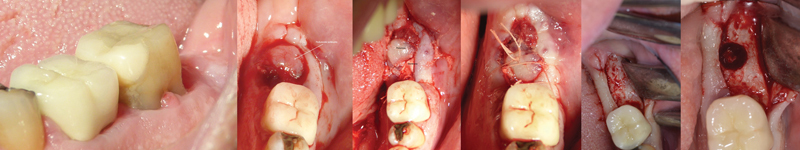
Group A: The granulation tissue was used to seal the defect together with a collagen membrane. The granulation tissue was detached to the socket and rotated to cover and close the occlusal socket hole using a surgical resorbable suture 5.0.

The pedunculated granulation tissue was used to seal the defect ( Fig. 1 ) together with a collagen membrane (Osseoguard, Zimmer Biomet dental, Palm Beach, Florida, United States).

The granulation tissue was detached to the socket and rotate to cover and close the occlusal socket hole using a surgical suture 5.0 (Vicryl, Ethicon). 23

#### Group B

**Fig. 2:**
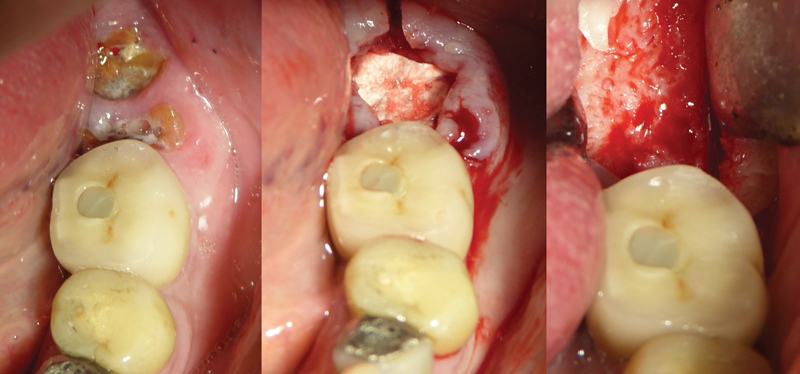
Group B: A collagen membrane was contoured into a modified V-shape that was used to seal the defect occlusally and was placed into the socket and should be wide enough to extend laterally past the defect in the buccal wall. The occlusal part of the membrane was attached to the gingiva using surgical resorbable sutures 5.0

A collagen membrane (Osseoguard, Zimmer Biomet dental, Palm Beach, Florida, United States) was contoured into a modified V-shape that was used to seal the defect occlusally and was placed into the socket; it should be wide enough to extend laterally past the defect in the buccal wall ( Fig. 2 ).

The occlusal part of the membrane was attached to the gingiva using surgical sutures 5.0 (Vicryl, Ethicon). 15

All extracted teeth belonged to molars or premolars and in all cases the regeneration surgery was performed immediately after the tooth extraction and tooth transforming procedures (about 30 minutes). No distinction was made among vital, nonvital tooth or tooth with endodontical root canal therapy because it was well demonstrated 15 that there are no significant differences using the following protocol for tooth transformation. The periodontal teeth were included, and, in most cases, they had the pediculated tissue used to cover the graft (in group A).

The extracted tooth was accurately cleaned from residual calculus and thoroughly polished by using a diamond drill (ref. 6855—Dentsply Maillefer) with abundant saline solution irrigation. The procedure required the complete remotion of any root filling material from the selected tooth. Afterward, the tooth was cut in small pieces and the fragments were placed in the mill (Tooth Transformer, Tooth Transformer srl, Milan, Italy) previously prepared, following the procedure described in previously published studies. 24 After 25 minutes, the material was ready to be placed in the socket preservation area. The graft is pressed into the socket and covered follow the two groups' indications.

Patients were instructed to keep oral hygiene, limiting to soft brushing for the first 2 weeks around the surgical site and rinsing twice a day with 0.12% chlorhexidine. 25

Dental implants were placed in the grafted area after a healing period of 4 months.

All patients underwent to CBCT scan of the grafted socket after 4 months of healing, before the implant surgery. The comparison between the initial CBCT scan (before the tooth extraction) and the second one (after 4 months of healing) allowed to assess the bone dimensional change from a radiographic point of view.

For the preparation of implant site, a 3 mm trephine bur was used under saline solution to collect a histological sample. After that a standard drill implants protocol was used to insert the implants.

The specimens were decalcified and the paraffin-embedded and cut. Samples were fixed in 10% neutral buffered formalin (37% formaldehyde solution, 10 mL; NaCl, 0.8 g; potassium phosphate monobasic, 0.4 g; potassium phosphate dibasic, 0.65 g; distilled water, 90 mL) for 7 days. Decalcification was carried out with disodium ethylenediaminetetraacetic acid pH 7 until total decalcification, and the end-point was determined physically. Samples were then dehydrated in ethanol of rising concentration from 70 to 100%, cleared with xylene, and embedded in paraffin; all the chemical use were manufactured from Carlo Erba reagents. Paraffin slides were obtained with a Leica RM2245 rotatory microtome and placed on superfrost microscope glass slides mounted with Biomout HM bio-optica. The histological images obtained from the transmitted light microscope (Olympus) were digitized through a digital camera and analyzed by means of an image analysis software IAS 2000 (QEA). The median section of each sample was split in nine subsections in order to evaluate if differences existed between different portion of the samples (lateral ones versus central ones or upper vs bottom). With histomorphometric analysis, we have distinguished:

- Bone volume% (BV%) that represented the percentage of mineralized tissue with exclusion of medullary tissues.
- Tooth transformer% (TT%) that represented the percentage of the volume occupied by the remaining graft granules, namely dentin.
- Vital bone% (VB%) that represented the percentage of vital bone, excluding medullary tissues.

The amount of BV% was the sum of TT% and VB%. Each subsection was measured using ImageJ software.

## Results

All the 16 patients showed good clinical healing of hard and soft tissues and in all site, it was possible to insert a standard titanium implant after 4 months. The physiologic bone resorption after tooth extraction appears minimum with a good maintenance of hard tissue architecture. No complications, such as suppuration or bleeding, occurred during the healing period. No clinical differences of the healing were detected between age, sex, or reason of tooth extraction. The success rate, corresponding to maintain the soft and hard tissue and to be able to insert implants, was the 100% of the cases. The comparison between the initial CBCT scan, before tooth extraction, and final CBCT scan, after 4 months of healing revealed the regeneration of the buccal wall and horizontal bone stability.

Sixteen histological samples were collected and the histomorphometrical results showed that group A had 46.1 ± 8.07 of BV%, 9.2 ± 9.46 of TT%, and 35.2 ± 12,36 of VB%, while in group B the BV% was 41.22 ± 5.88%, the TT% was 7.94 ± 7.54, and the VB% was 31.7 ± 7.52. The *t* -test revealed no significant differences between groups ( *p* > 0.05). All histomorphometrical data are summarized in Tables 1 2 3 .

**Table 1:** Bone volume percentage (BV%) values in all cases in both groups. No statistical differences between groups ( *p* > 0.05)

Bone volume%	Group A	Group B
Patient 1	46.56	38.13
Patient 2	35.02	46.64
Patient 3	39.42	34.65
Patient 4	48.04	36.15
Patient 5	56.23	35.14
Patient 6	48.69	48.88
Patient 7	38.15	43.04
Patient 8	56.69	47.22
Average	46.1 ± 8.07	41.22 ± 5.88

**Table 2:** Residual graft percentage (TT%) values in all cases in both groups. No statistical differences between groups ( *p* > 0.05)

Residual graft%	Group A	Group B
Patient 1	4.44	5.07
Patient 2	7.84	1.59
Patient 3	8.45	4.57
Patient 4	31.35	0.84
Patient 5	5.77	4.62
Patient 6	5.49	7.52
Patient 7	10.33	20.67
Patient 8	0	18.68
Average	9.2 ± 9.46	7.94 ± 7.54

**Table 3:** Vital bone percentage (VB%) values in all cases in both groups. No statistical differences between groups ( *p* > 0.05)

Vital bone%	Group A	Group B
Patient 1	42.11	33.05
Patient 2	27.17	43.37
Patient 3	29.97	30,8
Patient 4	16.69	23.64
Patient 5	38	30.52
Patient 6	43.2	41.35
Patient 7	27.82	22.33
Patient 8	56.69	28.55
Average	35.2 ± 12.36	31.7 ± 7.52

**Fig. 3:**
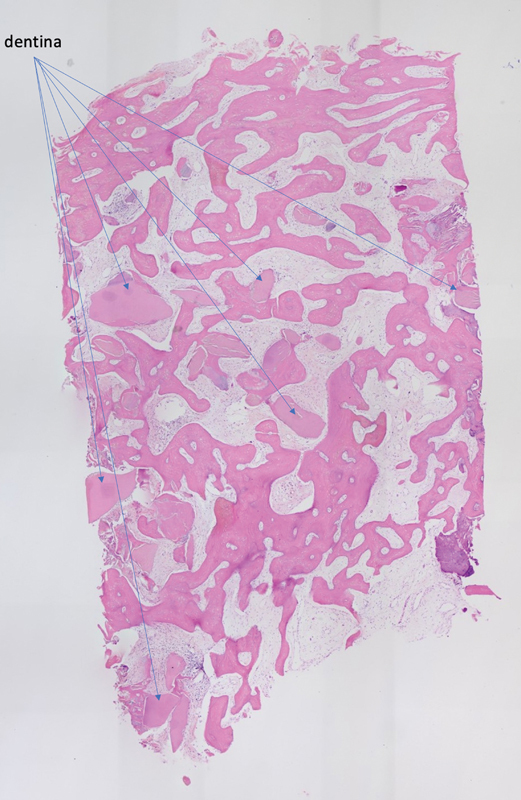
Group A. Histological analysis. A complete graft integration without any presence of inflammatory of connective tissue was evident. High percentage of new bone formation. Bone trabeculae are well interconnected.

**Fig. 4:**
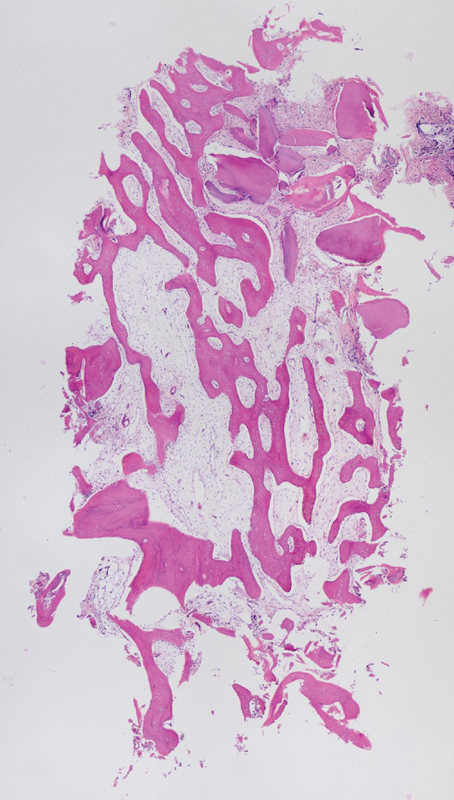
Group B. Histological analysis. New bone formation is visible. A complete graft integration without any presence of inflammatory of connective tissue was evident. Big spaces between bone trabeculae are evident.

The average quality of the bone appeared better using a pedunculate tissue ( Fig. 3 ) as compared to the ice cone technique ( Fig. 4 ).

In all cases, after implants insertion, complete osseointegration after proper healing period was achieved. After the healing period, hard and soft tissues were stable.

The healing of soft tissues after grafting procedures was particularly free of complications.

## Discussion

The soft and hard tissue preservation in post-extractive sites guarantees the long-term success of the rehabilitation in terms of esthetical, functional, and phonetical. 26 27 28 29 Alveolar ridge preservation techniques result in a significant reduction in the vertical bone dimensional change following tooth extraction when compared to unassisted socket healing. 30

The healing processes of the post-extraction sites after bone grafting procedures have been widely studied by many clinical trials analyzing different graft materials. 30 31 32 33 The studies agreed in recommending a post-extraction alveolus grafting procedure to reduce the bone resorption. A recent clinical trial comparing guided bone regeneration (GBR) versus socket seal technique revealed that GBR was more effective at reducing radiographic bone resorption. 34

To date, mainly bone of deproteinized bovine origin or alloplastic materials was used as graft material. 35 These materials showed the features of space maintaining and were embedded in the bone matrix in formation. The newly formed bone tissue will consist mainly of residues of the graft material and a small percentage of new bone. 36

On the other hand, the use of autologous bone as graft material showed the best results for its osteoconduction and osteoinduction properties. 37 38 However, all autologous bone grafting procedures required a second surgical site for bone harvesting or a double surgery treatment with increased discomfort of the patient. 39 To limit the discomfort, several biomaterials with slow or rapid reabsorption were suggested; however, all materials showed only osteoconductive capabilities. 40 41

Extracted teeth have been considered waste materials for a long time because it was ignored that dentin is chemically and physically very similar to bone with the only difference being that the latter is less mineralized. Furthermore, autologous demineralized dentin represents a source of autogenous growth factors for bone regeneration. 42 Nowadays, the autogenous tooth could be considered as a novel and innovative graft material. 43 The strong points in favor of using such material are it is totally autogenous, it does not require an additional surgical site for harvesting bone graft, the dentin structure and composition are very similar to that of bone, it has been shown to contain BMP-2, that is made available by the demineralization procedure, giving to the material with interesting properties. 44 The unmineralized autogenous dentin graft showed bone formation capacity on early period of bone healing of post-extractive socket in a recent randomized clinical trial. 45

A recent overview study 46 of tooth grafts confirmed that autogenous tooth bone grafts appear to be effective in oral defect reconstructions compared to Bio-Oss substitutes.

The purpose of the present study was to make a histological and clinical comparison between two different socket preservation approaches using a dentin graft material produced by the innovative device tooth transformer. This device, in fact, is totally automated and it can autonomously manage the shredding, disinfection, and demineralization phases. Many clinical studies, in which was used this device to obtain autologous particle dentin graft, showed good histomorphometric and histologic results in bone regeneration. In 2022, a histological study was presented to explore the histomorphometric outcomes of tooth derived materials used as bone substitute material in socket preservation procedures. 47

Outcomes of the present study showed high percentage of new bone formation (>40%) and low rates of residual dentin particles (<10% in most cases)

It is possible to note that the average quality of the bone is better using a pedunculate tissue as compared to the ice cone technique. The volume occupied by new bone trabeculae appeared greater in the group A in respect with group B, although statistical differences were found between group, probably due to the low sample size. A recent pilot prospective cohort study 48 confirmed that the periodontal granulation tissue preservation could be helpful in periodontal surgical procedure to perfect close the surgical wound. Probably the primary wound closure with the pedunculate tissue has determined the differences. However, the two surgical procedures used are as an alternative to each other depending to the presence of pediculate inflammatory tissue.

## Conclusion

The clinical and histological results of these surgical techniques combined with autologous dentin graft materials tooth derived showed predictable outcomes in terms of clinical bone volume and new vital bone volume.

Autogenous dentin graft with both flapless techniques has showed promising results with a high percentage of new vital bone around the residual graft material.

This suggested that the autogenous demineralized tooth graft obtained by the TT Transformer medical device can be considered a feasible, safe, and biocompatible alternative to other xenogeneic allogeneic biomaterials currently used in human alveolar socket augmentation procedures. If it is possible, it is recommended to use the pedunculate tissue to close the post-extractive socket.
